# Placental-Derived Biomaterials and Their Application to Wound Healing: A Review

**DOI:** 10.3390/bioengineering10070829

**Published:** 2023-07-12

**Authors:** Nicole M. Protzman, Yong Mao, Desiree Long, Raja Sivalenka, Anna Gosiewska, Robert J. Hariri, Stephen A. Brigido

**Affiliations:** 1 Healthcare Analytics, LLC, 78 Morningside Dr., Easton, PA 18045, USA; 2 Laboratory for Biomaterials Research, Department of Chemistry and Chemical Biology, Rutgers University, 145 Bevier Rd., Piscataway, NJ 08854, USA; 3 Research & Development, Degenerative Diseases, Celularity Inc., 170 Park Ave., Florham Park, NJ 07932, USAraja.sivalenka@celularity.com (R.S.);

**Keywords:** biomaterials, decellularization, extracellular matrix, placenta, placental-derived biomaterials, wound healing

## Abstract

Chronic wounds are associated with considerable patient morbidity and present a significant economic burden to the healthcare system. Often, chronic wounds are in a state of persistent inflammation and unable to progress to the next phase of wound healing. Placental-derived biomaterials are recognized for their biocompatibility, biodegradability, angiogenic, anti-inflammatory, antimicrobial, antifibrotic, immunomodulatory, and immune privileged properties. As such, placental-derived biomaterials have been used in wound management for more than a century. Placental-derived scaffolds are composed of extracellular matrix (ECM) that can mimic the native tissue, creating a reparative environment to promote ECM remodeling, cell migration, proliferation, and differentiation. Reliable evidence exists throughout the literature to support the safety and effectiveness of placental-derived biomaterials in wound healing. However, differences in source (i.e., anatomical regions of the placenta), preservation techniques, decellularization status, design, and clinical application have not been fully evaluated. This review provides an overview of wound healing and placental-derived biomaterials, summarizes the clinical results of placental-derived scaffolds in wound healing, and suggests directions for future work.

## 1. Introduction

Complex, hard-to-heal wounds present a significant clinical challenge and are associated with considerable patient morbidity. Often, chronic wounds are unable to progress past the inflammatory phase of wound healing. Consequently, the wound is burdened by elevated concentrations of pro-inflammatory cytokines and imbalanced proteolytic enzymes and protease inhibitors, resulting in high concentrations of matrix metalloproteinases (MMPs), which destroy the extracellular matrix (ECM) [1,2].

A promising strategy for the treatment of nonhealing wounds is the application of placental-derived biomaterials. Placental-derived biomaterials are known for their biocompatibility, biodegradability, and low immunogenicity [3], making them ideal for use in medical applications. Research has shown that placental-derived biomaterials can be used to promote wound healing by providing an ECM scaffold for tissue repair [4], exerting anti-inflammatory effects [4,5,6], facilitating cell migration [5,6], and promoting regeneration [7].

This review provides an overview of wound healing and placental-derived biomaterials, summarizes the clinical results of placental-derived scaffolds in wound healing, and presents directions for future work. The focus of this review is the clinical application of placental-derived scaffolds in wound healing. Although placental-derived cell-based therapy shows promising clinical application, it is beyond the scope of this review.

## 2. Overview of Wound Healing

**Figure 1:**
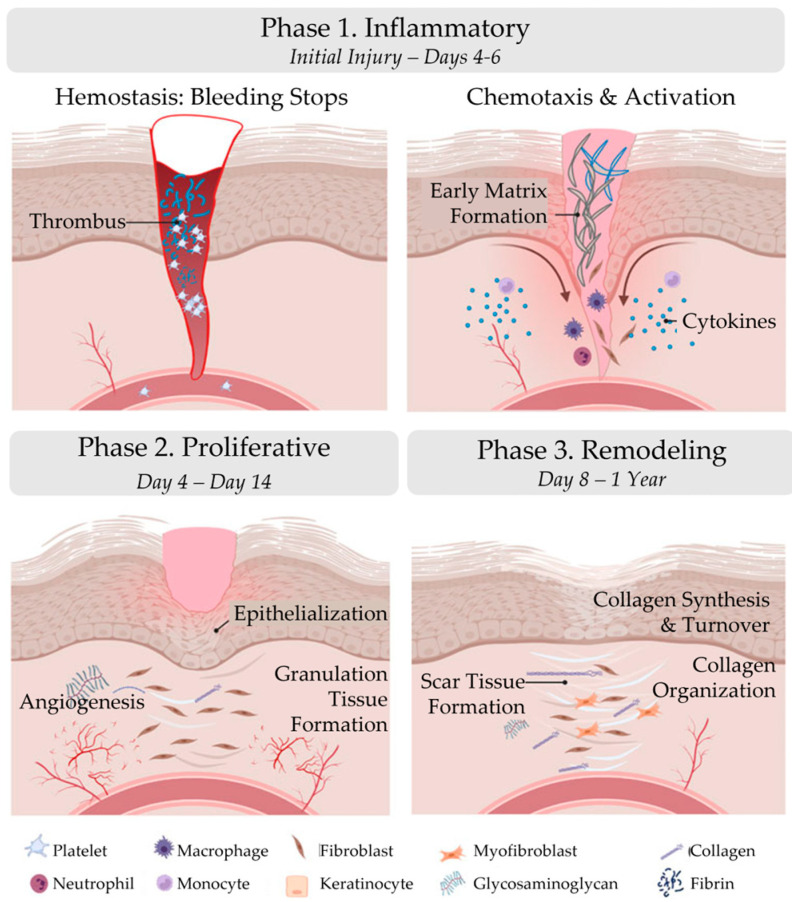
Phases of wound healing. Wound healing is divided into three distinct phases: inflammatory, proliferative, and remodeling. The figure was originally published by Frontiers under the Creative Commons Attribution License (CC-BY), permitting unrestricted use [9]. The figure has been adapted.

Wound healing is the process by which the body repairs and regenerates damaged tissue after an injury. It is a complex and dynamic process that involves a variety of cellular and molecular events, including homeostasis, inflammation, cell migration, proliferation, and tissue remodeling. Wound healing is divided into three distinct phases: inflammatory, proliferative, and remodeling (Figure 1; Table 1) [8].

### 2.1. Inflammatory Phase

The inflammatory phase of wound healing includes hemostasis and inflammation. Immediately following injury to the skin the inflammatory phase is initiated. Wound formation activates a clotting cascade, involving the temporary release of vasoconstrictors to reduce bleeding, and a fibrin clot is formed. The fibrin clot consists of collagen, platelets, thrombin, and fibronectin, which stimulate the release of cytokines and growth factors, such as interleukin-1 (IL-1), tumor necrosis factor alpha (TNF-α), transforming growth factor beta, and platelet factor-4 [8]. The fibrin clot serves as a scaffold for infiltrating cells, such as neutrophils, monocytes, fibroblasts, and endothelial cells, and concentrates the growth factors and cytokines [8,10]. After vasoconstriction, vasodilation occurs, which causes hyperemia and edema [8]. The released chemotactic factors and growth factors complete hemostasis and start inflammation [10].

Neutrophils are first recruited to the wound area and initiate phagocytosis. These cells release reactive oxygen species, proteases, and other enzymes that help debride the wound and remove any debris or bacteria. In addition, these cells release chemokines which serve as chemoattractants for other cell types and release pro-inflammatory cytokines. Next, leukocytes, including monocytes, are active in the wound area.

Macrophages are a type of white blood cell that play a key role in the body’s healing response [11]. Approximately 48–96 h after injury, monocytes differentiate into macrophages [8]. During wound healing, macrophages respond to temporal and spatial cues in their environment by altering their phenotypic polarization [12,13,14]. The M1 phenotype initiates the inflammatory response [10], and in the later phases of wound healing, the M1 phenotype transitions to the M2 phenotype, which facilitates tissue remodeling, repair, and the resolution of the healing process [15,16,17]. In chronic wounds, macrophages are believed to persist in an uncontrolled, pro-inflammatory (M1) activation state [1].

### 2.2. Proliferative Phase

The proliferative phase of wound healing is the building phase, characterized by epithelialization, angiogenesis, and granulation tissue formation. Fibroblasts and endothelial cells are the primary proliferating cells during this phase. Epithelialization begins as soon as the wound occurs and is stimulated by inflammatory cytokines and growth factors [8,18]. Keratinocytes are stimulated to migrate into the wound area, proliferate, and differentiate into the epidermis. Angiogenesis occurs simultaneously and is stimulated by local hypoxia, vascular endothelial growth factor, platelet-derived growth factor (PDGF), fibroblast growth factor-basic, and the serine protease thrombin [10,18,19]. Angiogenesis is marked by capillary formation and endothelial cell migration [8]. Lastly, granulation tissue formation occurs. Fibroblasts migrate to the wound site from adjacent tissues and proliferate [8,10,18]. In response to PDGF, fibroblasts synthesize a provisional matrix, consisting of type III collagen, glycosaminoglycans, and fibronectin [8].

### 2.3. Remodeling Phase

During the remodeling phase, granulation tissue formation ends, and wound maturation begins. This phase is characterized by the reorganization of collagen fibers and the contraction of the wound edges. The new tissue is remodeled and organized in an orderly manner to strengthen the repair [18]. Type III collagen is replaced with type I collagen [18], increasing the tensile strength of the wound. Matrix remodeling enzymes, particularly MMPs, play an important role in the remodeling of the local wound environment, as they break down ECM matrix components, such as collagen and elastin, allowing for tissue remodeling and the formation of new blood vessels. Even after a year, however, the wound tissue does not achieve the same strength as that of collagen from uninjured skin due to the formation of scar tissue.

### 2.4. Wound Formation

**Figure 2:**
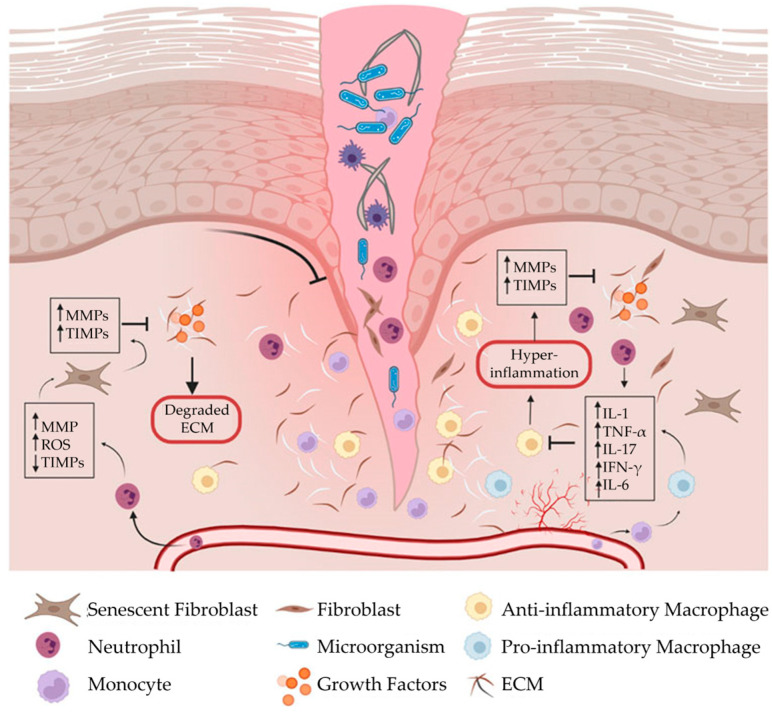
Chronic wound formation. When the phases of wound healing are disrupted, abnormal healing occurs, and the wound fails to proceed through a normal, orderly, and timely sequence of repair. Often, chronic wounds remain in a prolonged state of persistent inflammation and are characterized by elevated concentrations of pro-inflammatory cytokines, pro-inflammatory (M1) macrophages, and proteases, which destroy the extracellular matrix. In normal wound healing, the extracellular matrix regulates macrophage behavior. In a chronic wound, however, a vicious cycle exists between a dysfunctional extracellular matrix and uncontrolled M1 macrophages. ECM, extracellular matrix; IFN-γ, interferon gamma; IL-1, interleukin-1; IL-6, interleukin-6; IL-17, interleukin-17; MMPs, matrix metalloproteinases; ROS, reactive oxygen species; TIMPs, tissue inhibitors of metalloproteinases; TNF-α, tumor necrosis factor-alpha. The figure was originally published by Frontiers under the Creative Commons Attribution License (CC-BY), permitting unrestricted use [9].

When the phases of wound healing are disrupted, abnormal healing occurs, and a chronic wound can develop. A chronic wound is a wound that fails to proceed through a normal, orderly, and timely sequence of repair within an expected timeframe, usually 3 months or longer [20,21]. Often, these wounds remain in a prolonged state of persistent inflammation and are unable to progress to the next phase of wound healing (Figure 2; List 1) [19]. This state of persistent inflammation is characterized by elevated pro-inflammatory cytokines, dysfunctional macrophages, high protease concentrations, and an abnormal ECM. In normal wound healing, the ECM regulates macrophage behavior. However, in a chronic wound, a vicious cycle exists between an abnormal ECM and uncontrolled M1 macrophages.

List 1. Characteristics of persistent inflammation [1,2]:

- Elevated pro-inflammatory cytokines;
- Dysfunctional macrophages;
- Imbalanced proteolytic enzymes and protease inhibitors;
- High concentrations of MMPs;
- Abnormal ECM.

### 2.5. Chronic Inflammation Leading to Fibrosis

Chronic inflammation plays a crucial role in the activation of fibrosis or scarring. When wound healing becomes dysregulated, collagen deposition can devolve into an irreversible accumulation of fibrotic connective tissue by ECM-producing myofibroblasts [22]. As highlighted by Wynn and Ramalingam [22], there are many underlying mechanisms and mediators that contribute to the pathogenesis of fibrosis. For example, during the inflammatory phase of wound healing, neutrophils are recruited to the wound area to initiate phagocytosis. While these cells release reactive oxygen species, proteases, and other enzymes that help debride the wound and remove any debris or bacteria, any prolonged disturbance could lead to excessive tissue damage, leading to fibrosis [22].

### 2.6. Factors That Impair Wound Healing

Impaired healing and wound formation occur when the body’s natural healing process is disrupted or compromised [23]. The main risk factors that can lead to chronic wound formation can be grouped into local factors and systemic factors (Table 2) [24]. These factors are often interrelated with systemic factors acting locally to influence wound healing [24]. Local factors include oxygenation, infection, and venous insufficiency [19,24,25]. Systemic factors include age, ambulatory status, comorbidities, medications, oncology interventions, and lifestyle habits [19,24,25]. When the healing process is impaired, wounds may take longer to heal or may not heal at all, which increases the risk of complications, such as infection, scarring, and even amputation. Effective treatment may involve addressing the underlying cause of the problem, as well as appropriate wound care to support healing and prevent further complications.

Many patients who experience chronic wounds have underlying medical conditions, such as diabetes, venous insufficiency, or obesity. The most common types of chronic wounds include arterial ulcers, diabetic ulcers, pressure ulcers, and venous ulcers [20,21].

### 2.7. Economic Impact of Chronic Wounds

It has been estimated that 1–2% of the general population in developed countries will experience a chronic wound [26,27]. In the United States, chronic nonhealing wounds impact 8.2 million Medicare beneficiaries with associated costs ranging from USD 28.1 to USD 96.8 billion [23]. The alarming number of patients affected by chronic nonhealing wounds is expected to rise because of the combined effects of an aging population and the rising rates of diabetes and obesity [28,29,30]. As such, chronic wounds represent a significant economic burden to the healthcare system [31].

### 2.8. Treatment of Chronic Wounds

The basic tenets of wound care follow the TIME principle: tissue debridement, infection control, moisture balance, and edges of the wound [20,32]. Once standard measures have been taken, an ulcer must be diagnosed, and treatment tailored to the specific type of ulcer. Those with arterial ulcers should be directed to a vascular surgeon for immediate attention [20]. Venous ulcers require compression, elevation of the lower limbs, and exercise if possible [20,33]. Diabetic foot ulcers require offloading and, if necessary, treatment of any underlying peripheral arterial disease [20,34,35]. Pressure ulcers should be managed with a repositioning schedule to reduce pressure on the affected area [20,36,37].

While the TIME principle remains a mainstay of treatment, several additional therapies have been suggested to improve wound healing (List 2).

List 2. Additional wound healing strategies:

- Negative pressure wound therapy [38,39];
- Hyperbaric oxygen therapy [40,41,42,43];
- Autologous platelet-rich plasma [44,45];
- Growth factors [46,47];
- Cell therapy [48,49];
- Scaffolds (e.g., autologous, biologic, and synthetic) [50,51,52,53].

## 3. Placental-Derived Biomaterials

### 3.1. Source of Placental-Derived Biomaterials

**Figure 3:**
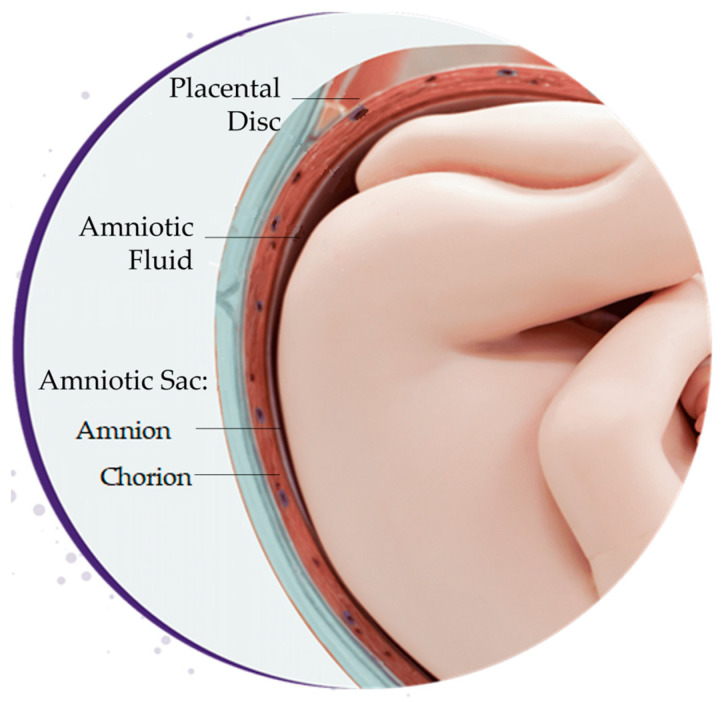
Sources of placental-derived biomaterials. Placental-derived biomaterials can be sourced from the amniotic sac, amniotic fluid, placental disc, umbilical cord, or a combination of these sources. The amniotic sac is composed of the amnion and chorion layers.

The placenta is a vital temporary embryonic and later fetal organ that connects the developing fetus to the maternal uterine wall in humans. Placental-derived biomaterials, also referred to as perinatal derivatives, include different placental tissues, sourced from the amniotic sac, amniotic fluid, placental disc, umbilical cord, or a combination of these sources [54,55] (Figure 3). This includes not only the tissues themselves but also the entities within these structures, including fluids, gels, and cells [55,56].

#### 3.1.1. Amniotic Fluid

The amniotic fluid surrounds the embryo and fetus during development and has a myriad of functions [57]. The amniotic fluid contains a variety of nutrients and growth factors that facilitate fetal growth, provides physical protection by cushioning the fetus and umbilical cord, and has antimicrobial properties, which protect the fetus from infection. The amniotic fluid is primarily composed of water and electrolytes, with signaling molecules, peptides, carbohydrates, lipids, proteins, and hormones making up a small percent [58,59]. Hyaluronic acid is also suspended within the amniotic fluid, increasing the viscosity [60].

#### 3.1.2. Amniotic Sac

**Figure 4:**
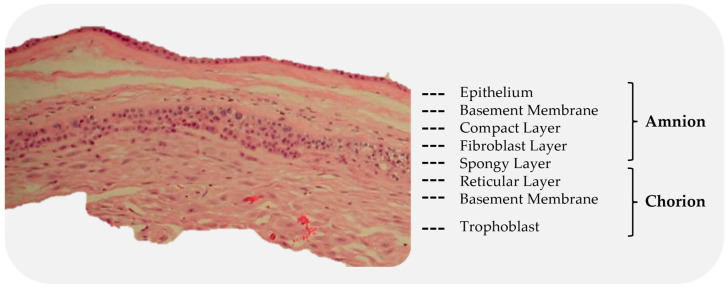
Subdivision of the amnion and chorion layers. The red structures, visible in the chorion, are blood vessels.

The amniotic sac is a thin semi-transparent membrane that holds the amniotic fluid for the developing fetus [61]. It is composed of an avascular layer, the amnion, and a highly vascularized layer, the chorion (Figure 4) [61,62].

**Figure 5:**
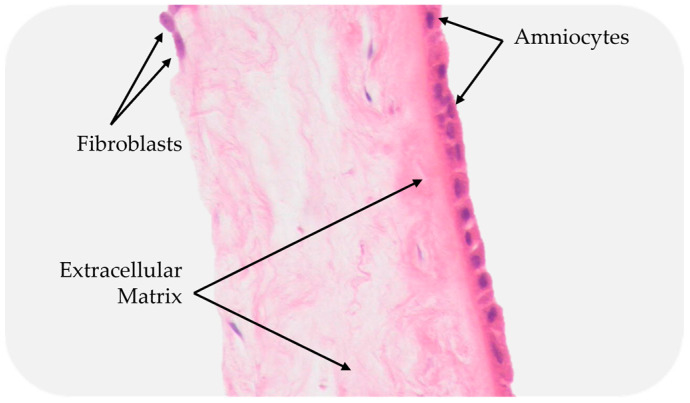
Cross-section of a placental membrane with the chorion layer removed.

The amniotic membrane (AM) is the innermost of the two membranes, delimiting the amniotic cavity and bathed in amniotic fluid [63]. The AM measures 0.02 mm–0.05 mm in thickness and has a multilayered architecture: an epithelium, a basement membrane, and a collagen-rich stromal layer. The epithelium is a monolayer of metabolically active cuboidal cells with microvilli present on the apical surface. The basement membrane is one of the thickest membranes in the human body [3]. It is made up of a rich collagen framework in addition to bioactive molecules, such as fibronectin and laminin [3,61]. The stromal layer can be further subdivided into three layers: a compact layer, a fibroblast layer, and a spongy layer. The compact layer provides the fibrous structure of the amnion [3]. Interstitial collagens (types I and III) form parallel bundles that provide mechanical integrity, while collagens type V and VI create filamentous connections between the interstitial collagens and the basement membrane [3,61]. The intermediate spongy layer is composed of a nonfibrillar meshwork of mostly type III collagen, as well as an abundance of proteoglycans and glycoproteins [3,64]. It loosely connects the amnion and chorion membranes, which allows the two membranes to be easily separated by blunt dissection [3] (Figure 5).

The chorion is the outermost membrane of the amniotic sac and is in contact with the amnion on the inner aspect and the maternal decidua on the outer [62]. Like the AM, the chorion membrane also has several layers: a reticular layer, a basement membrane, and a trophoblast layer. The reticular layer is the thickest layer of the chorion and is composed of collagen types I, III, IV, V, and VI and proteoglycans. The basement membrane is a dense layer of connective tissue (type IV collagen, fibronectin, and laminin) that adheres the trophoblasts and the reticular layer [62,64]. The trophoblast layer interfaces with the maternal decidua on the surface of the placental disc and consists of 2–10 layers of trophoblasts [61,62].

#### 3.1.3. Placental Disc

The placental disc provides a link between the developing fetus and the mother, regulating nutrition, waste removal, hormonal balance, and the immune system while also acting as an immunologically privileged barrier to prevent direct contact between their respective blood supplies [65]. The placental disc is composed of a highly vascularized ECM, containing collagen types I, III, IV, and VI, as well as a vast distribution of noncollagenous glycoproteins and proteoglycans, such as fibronectin, fibrillin I, laminin, thrombospondin I, tenascin C, decorin, heparan sulfate proteoglycans, and elastin [66]. Within the placental disc, a variety of cell types can be found, such as trophoblasts (i.e., syncytiotrophoblasts/cytotrophoblasts), mesenchymal cells, and mesenchymal-derived macrophages, fibroblasts, and fetal vascular cells (i.e., vascular smooth muscle cells, perivascular cells, and endothelial cells) [67]. In addition to the various cell types, the placental disc is also rich in nutrients and cytokines [61].

#### 3.1.4. Umbilical Cord

The umbilical cord contains three vessels, the umbilical vein and two umbilical arteries, which are embedded in Wharton’s Jelly and surrounded by a single epithelial layer, derived from the amnion [68,69]. The umbilical vein transports oxygenated blood from the placenta to the to the fetal heart, and the arteries return deoxygenated blood and waste away from the fetus and to the placenta [69]. Wharton’s Jelly is a mucoid connective tissue composed of a network of glycoprotein microfibrils and collagen fibrils [70]. Collagen types I, III, V, and VI have been identified in Wharton’s Jelly [71,72]. Hyaluronic acid, the most abundant glycosaminoglycan in Wharton’s Jelly [71], creates a hydrated gel around the fibroblasts and collagen fibrils, which maintains the architecture of the umbilical cord and provides protection from pressure [71,73,74]. The cell population of Wharton’s Jelly includes fibroblast-like cells, myofibroblast-like cells, and mesenchymal stem cells [73,75,76].

### 3.2. Properties of Placental-Derived Biomaterials

The human placenta is a temporary vital organ that is usually discarded as medical waste, making it an easily accessible, cost-effective, and ethical source of raw material. In addition to its availability, the placenta possesses several desirable biological properties that are innate to healing, including angiogenic, anti-inflammatory, antimicrobial, antifibrotic, and immunomodulatory with low immunogenicity (List 3). Complementary to the desirable biological properties, placental tissues have unique ECMs with notable structural and mechanical properties, including elasticity, stiffness, and tensile strength [77]. However, the ECM composition varies with the source, as described in the previous section.

List 3. Biologic properties of the placenta:

- Angiogenic [78];
- Anti-inflammatory [5,6,79,80,81];
- Antimicrobial [3,63,82,83,84];
- Antifibrotic [85,86];
- Immunomodulatory [4,87,88,89,90];
- Low immunogenicity [91].

In addition to the aforementioned properties, placental-derived biomaterials, containing viable cells, can also act through paracrine mechanisms [92]. The cells contained within placental-derived biomaterials stimulate tissue repair by mediating the release of trophic factors [93] and immunomodulation [94,95]. Moreover, the growth factors and cytokines released by placental-derived biomaterials facilitate anti-inflammatory and antimicrobial actions [96,97,98].

### 3.3. Differences among Placental-Derived Biomaterials

Despite a growing body of evidence demonstrating that placental-derived biomaterials have the capacity to enhance healing, the methods of processing and preparing the tissue are continually evolving. Variations in tissue source, preservation, decellularization, design, and application have the potential to affect the biological and mechanical characteristics of the tissue.

As previously noted, placental-derived biomaterials can be derived from a variety of sources, including the amniotic sac (e.g., amnion and chorion), amniotic fluid, and umbilical cord (e.g., umbilical cord blood, umbilical cord tissue, and Wharton’s Jelly) or a combination of these sources [54]. While this provides a plethora of biomaterials, it also introduces significant variability. Differences in composition exist among sources. For example, the AM has a collagen-rich ECM and contains several bioactive ECM molecules, such as fibronectin, laminin, elastin, and glycosaminoglycans [99], while Wharton’s Jelly is a mucoid connective tissue composed of a network of glycoprotein microfibrils and collagen fibrils [70]. In addition, research has shown that the immunomodulatory properties are source dependent [100]. Even when comparing biomaterials from the same source, interdonor and intradonor variability exists [101].

### 3.4. Preservation Method

Following delivery, the placenta is usually discarded as medical waste. Alternatively, placental-derived biomaterials can be obtained following normal, healthy, and full-term pregnancies. Comprehensive screening of the donors is required before the tissue is procured and processed. With appropriate consent, the placenta is collected after delivery and donated. After the tissue is collected, the placenta is transported for processing. Although the specific testing requirements may vary depending on local and regional guidelines, proper screening is required to test for infectious diseases, such as human immunodeficiency virus, hepatitis B virus, hepatitis C virus, West Nile, and syphilis. The tissue is sterilized to minimize the risk of disease transmission to recipients and is processed to allow prolonged storage. Tissue preservation is usually accomplished by one of several techniques, most commonly cryopreservation, dehydration, or lyophilization [63]. It is important to note that all methods of preservation compromise the tissue’s integrity to varying degrees.

Cryopreservation or freezing is the most widely used method of tissue preservation. Cryopreservation is a process in which the structure and function of cells, tissues, or organs are preserved by cooling the samples to very low temperatures [102,103]. As part of the cryopreservation process, cryoprotectants are mixed with cells and tissues to reduce ice crystal formation. Various cryoprotectants have been used to preserve placental-derived biomaterials, including glycerol, dimethylsulfoxide, and ethylene glycol. However, the cryopreservation method is criticized for impairing the viability and proliferative capacity of cells and requires the tissue to be shipped and stored at −80 °C [104].

Dehydration is an alternate method of tissue preservation that removes the water from the tissue sample. This process helps to preserve the structural and biochemical activity of placental tissue and prevent the growth of microorganisms and the breakdown of the tissue. Dehydration is achieved using air or heat to dehydrate the tissue [61]. Unlike cryopreservation, dehydration preserves the tissue without the need for freezers, dry ice, or liquid nitrogen and can be shipped and stored at room temperature with a 5+ year shelf-life. The dehydration of tissue provides an additional advantage of allowing allografts to be terminally sterilized, thus reducing the risk of infectious disease transmission from the donor tissue.

Lyophilization or freeze drying involves cooling the tissue to −80 °C and using a sublimation process to remove the water by vacuum desiccation [105]. Like cryopreservation, the freezing step in this process can cause ice crystal formation and damage to the tissues. To reduce tissue damage, sugars can be used as a cryoprotectant to stabilize the proteins [106]. Similar to dehydration, lyophilization allows tissues to be shipped and stored at room temperature without the need for freezers or liquid nitrogen [105].

### 3.5. Decellularization

**Figure 6:**
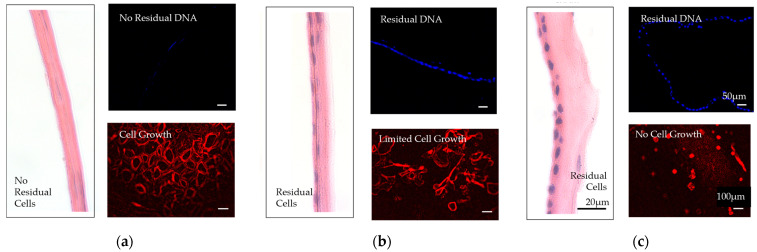
Comparison of decellularized and nondecellularized amniotic membranes. (**a**) A proprietarily processed decellularized dehydrated amniotic membrane (BIOVANCE^®^, Celularity Inc., Florham Park, NJ) is shown. The decellularization process completely removes the residual cellular components, cells, cells debris, and DNA, as well as growth factors and cytokines. The collagen framework remains intact in its native three-dimensional form with essential extracellular matrix molecules. (**b**) Nondecellularized dehydrated amniotic membrane. (**c**) Nondecellularized cryopreserved amniotic membranes are shown. These membranes retain residual cellular components, cells, cell debris, and DNA, as well as growth factors and cytokines. The images are used with permission from the original publisher [6]. Images were originally published by and used with permission from John Wiley and Sons.

Decellularization is a process by which the cellular components of a tissue or organ (e.g., endogenous cells, cell debris, and genetic materials) are removed, while the structural and regulatory proteins of the ECM are preserved (Figure 6).

This process is used to create acellular tissues and organs for use in regenerative medicine and tissue engineering applications. The elimination of cellular content from natural tissue-derived matrices has the potential to promote healing, integration with host tissues, and limit a foreign body reaction [107]. The decellularization process typically involves the use of chemicals, enzymes, physical forces, and/or a combination of these methods to remove cellular components, while preserving the ECM [108,109]. Methods of decellularization affect the structure of the ECM, the structure of the tissue, and the biomechanical behavior. Therefore, it is important to find methods that balance the removal of cellular content with the retention of the structures and entities within the ECM. Several methods have been used in the decellularization of placental tissues [9,109,110,111,112]. Table 3 summarizes the methods and agents used to achieve an acellular scaffold.

As indicated, tissue preservation and decellularization methods have specific advantages and disadvantages. The processing of placental tissues aims to remove any hazardous materials while preserving the structural and biochemical activity of the tissue to optimize healing.

### 3.6. Clinical Application

**Figure 7:**
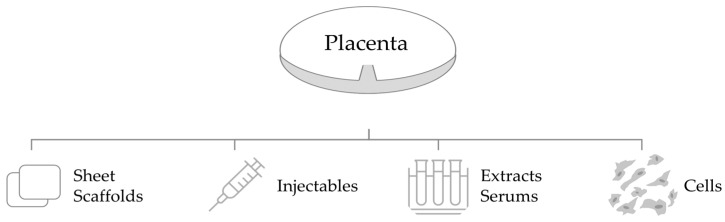
Forms of placental-derived biomaterials. Various forms of biomaterials are extracted from the placenta, including (1) sheet scaffolds; (2) injectables; (3) extracts/serums; (4) cells.

The clinical application of placental-derived biomaterials also varies in form, administration, and delivery system. Placental-derived biomaterials are available in many forms, including sheet scaffolds, injectables, extracts, and cells (Figure 7) [56]. Reported administrations include topical application, intradermal/subcutaneous injection, and intravenous or intraperitoneal injection [49]. Delivery systems include hydrogels, synthetic or natural biomaterials as carriers for transplanted cells, and extracts or secretomes [49].

Several cell types exist within the placenta and have different mechanisms of action, which are source dependent [100]. While the therapeutic benefit of placental-derived mesenchymal stem cells in wound healing has been described throughout the literature [125,126,127], it is not a subject of this review. Rather, this review focuses on the use of human placental-derived biomaterials as scaffolds for wound healing. Placental-derived biomaterials are available as scaffolds in two principal forms: sheets and injectables [128].

The human AM is one of the most widely used and studied placental-derived biomaterials. Human AM sheets are processed to retain the native ECM structure with its high collagen content and key bioactive molecules, such as fibronectin, laminin, glycosaminoglycans, and elastin [99]. Some scaffolds undergo proprietary processing procedures to retain the native growth factors and cytokines [99], while others are decellularized to remove all residual cells, cell debris, growth factors, and cytokines [129]. Although sheet scaffolds are commonly used as a wound covering, they can also be secured to the wound bed, permitting application to wounds of varying severity [117]. Sheet scaffold can be composed of amnion alone [130,131], amnion and chorion [132,133,134,135,136], umbilical cord alone [136,137,138,139,140,141], or umbilical cord and amnion [142]. The sheet ECMs possess the properties associated with their tissue of origin [56]. For example, AM sheet scaffolds are thought to promote healing via epithelialization [143,144,145], reduction of inflammation [6,79,80,81], inhibition of scar tissue formation [85,86], and the ability to act as an antimicrobial agent [82,83,84]. In addition, sheet scaffolds are modified to improve the mechanical properties of the tissue and are available in many configurations, including single layer and multilayer [6], full-thickness, and composite grafts. For example, lamination of sheet scaffolds is performed to create biomaterials with improved handleability and tensile strength [6,146].

Placental-derived biomaterials are also available in an injectable form as suspension allografts [147] and particulates/micronized powders [128,148,149]. The micronized powders can be applied directly to the wound or can be rehydrated and injected through a syringe directly into the wound until approximately one- to two-thirds of the wound is filled [128,148]. Commercially available products are sourced from the amnion, chorion, amniotic fluid, umbilical cord, and placental disc or a combination of these sources. Similar to the sheet scaffolds, the micronized form provides a connective tissue matrix complete with regulatory proteins, which stimulate cell migration [5], proliferation [5], and epithelialization [150,151,152]. Unlike the sheet scaffolds, the injectable form offers the added benefits of filling irregularly shaped and deep tunneling wounds and is reportedly easier to handle intraoperatively [128,149,153].

### 3.7. Commercial Products

Placental-derived allografts are processed from human tissue according to the American Association of Tissue Banks (AATB) standards and are regulated as a Human Cell, Tissue, or Cellular or Tissue-Based Product (HCT/P) by the US FDA under section 361 of the Public Health Service act as HCT/P (21 CFR, Part 127.10a). According to these guidelines, at a minimum, these products must be minimally manipulated, not combined with drugs or devices, and not reliant on cell metabolic activity as a primary function. In 2017, the company AmnioChor provided a list of placental-derived tissue products sold under section 361 [154]. In total, 116 products were listed. Table 4 and Table 5 provide an updated list of the commercially available placental-derived scaffolds, intended for wound healing applications.

This list is not exhaustive and, therefore, does not represent all commercially available HCT/Ps under section 361. However, it does provide a scope of the available products and variations in source, preservation methods, decellularization status, and unique design characteristics, where applicable.

## 4. Placental-Derived Biomaterials in Wound Healing: Clinical Results

Given the innate healing properties of the placenta, placental-derived biomaterials have been investigated as advanced wound care therapies for more than 100 years. The first clinical application of placental-derived biomaterials was reported in 1910, when Davis used AM as a substrate for skin transplantation [179]. At that time, only fresh AM was available, which was difficult to procure and carried a significant risk of disease transmission. With advancements in tissue processing and preservation, the use of these biomaterials has expanded considerably and now includes applications in tissue engineering, regenerative medicine, and cell-based therapies [3,148].

### 4.1. Outcomes

Randomized controlled trials (RCTs) provide the most reliable evidence for determining the effectiveness of a treatment/intervention. A comprehensive literature search was performed to identify RCTs published in the last ten years that evaluated the application of placental-derived scaffolds to treat nonhealing wounds. The PubMed database was queried for the terms “placenta matrix wound healing”, “amnion wound healing”, “chorion wound healing”, “umbilical cord wound healing”, and “amniotic fluid wound healing”. Inclusion criteria included randomized controlled trials reporting on the use of placental-derived scaffolds to treat nonhealing wounds, clinical outcomes, and human subjects. Exclusion criteria included animal data, basic science studies, review articles, articles with inadequate sample sizes for statistical analysis, studies evaluating skin grafts, burn wound healing, punch biopsy wounds, and non-English language literature. The search was limited to the previous 10 years. A summary of the results is provided in Table 6.

Although a large percentage of the randomized controlled trials published within the last 10 years focused on the application of dehydrated human amnion/chorion membranes (dHACMs), the literature also includes studies evaluating the application of dehydrated amnion powder, dried human AM, hypothermically stored AM, and umbilical cord allografts in the treatment of hard-to-heal ulcers. The research consistently demonstrates the effectiveness of placental-derived biomaterials to treat diabetic foot ulcers (DFUs) and venous leg ulcers (VLUs). Several of these high-level studies compared the application of placental-derived biomaterials with standard wound care and demonstrated that the application of placental-derived biomaterials significantly improves the proportion of healed ulcers [132,135,137,171,175,181,184,185,186,187,189,190], time to healing and rates of healing [132,135,137,175,184,185,186,187,191], and ulcer size [171,180,182,189,191]. In addition, Selena and colleagues [190] reported that 79.5% of patients treated with dHACM and multilayer compression therapy reported reduced VLU pain, compared with 52.4% patients who were treated with multilayer compression therapy alone. This finding suggests that the application of placental-derived biomaterials may also alleviate the pain associated with VLUs.

To better understand the ideal application strategy, two studies evaluated the frequency of placental-derived biomaterial application [189,190]. In 2014, Serena and colleagues [189] conducted a multicenter study evaluating the use of dHACM and multilayer compression therapy versus multilayer compression therapy alone in the treatment of VLUs. As a secondary aim, the study compared the proportion of VLUs demonstrating ≥40% closure at 4 weeks in patients receiving one application of dHACM versus two applications of dHACM. After 4 weeks, the proportion of wounds demonstrating a ≥40% closure was similar with one or two applications (62% and 63%, respectively). However, the lack of a significant difference in dHACM application frequency may be attributable to the short study period of 4 weeks. In 2014, Zelen and colleagues [190] conducted a similar study over 12 weeks to determine if the weekly application of dHACMs reduces the time to healing more effectively than biweekly applications for the management of DFUs. Although the proportion of DFUs that achieved complete healing were similar between the two groups, DFUs receiving weekly application of dHACM healed significantly faster than those receiving biweekly dHACM applications. These results suggest that more frequent application reduces the time to healing. However, additional studies are needed to determine the optimal application frequency for placental-derived biomaterials.

Given the significant economic burden associated with treating DFUs and VLUs, investigators have analyzed the economic impact of treating ulcers with placental-derived products [183,185,186,187]. In each of these analyses, the results demonstrate that the application of placental-derived biomaterials is a cost-effective treatment for ulcers. For example, in 2015, Zelen and colleagues [187] conducted an interim analysis of 60 patients and compared the application of Apligraf^®^ (Organogenesis, Inc., Canton, MA, USA) and EpiFix^®^ (MiMedx Group Inc.,Marietta, GA, USA) for the treatment of DFUs. The study found that the application of dHACM significantly reduced the median number of grafts (2.15 vs. 6.2 grafts), as well as the median graft cost per healed wound (USD 1669 vs. USD 9216). The study was continued, expanding the cohort to 100 patients and, again, dHACM was found to significantly reduce the median number of grafts (2.5 vs. 6 grafts) and the median graft cost per healed wound (USD 1517 vs. USD 8918) [186]. Using the data from a previously published study by DiDomenico and colleagues [185], Carter [183] conducted an economics health study to estimate the cost-utility of an aseptically processed dehydrated human amnion and chorion allograft (dHACA) (AmnioBand^®^, Musculoskeletal Transplant Foundation (MTF), Edison, NJ, USA) plus standard wound care versus standard wound care alone. This data modeling study demonstrated that the use of a dHACA combined with standard wound care compared with standard wound care alone is a cost-effective treatment for DFUs. When collectively considered, these results confirm the superior resource utilization with the application of placental-derived biomaterials.

RCTs have been criticized for excluding a large percentage of the population because of strict inclusion and exclusion criteria and, therefore, not generalizing to the population at large [130]. Although not randomized or controlled, in 2015, Smiell and colleagues [130] conducted a real-world multicenter trial, evaluating wound closure, following treatment of uninfected full- and partial-thickness wounds with a decellularized dehydrated human amniotic membrane (DDHAM). Chronic wounds included venous ulcers, diabetic ulcers, pressure ulcers, arterial ulcers, and collagen vascular ulcers of varying severity and duration. In total, 179 wounds in 165 patients were included. The two most common ulcer types were venous ulcers (50%) and diabetic ulcers (26%). The median time to closure in the Good Wound Care group, a subset of compliant patients, was 6.3 weeks. Most notably, 50% of patients who had failed treatment with one or more advanced biologic therapies achieved complete closure after treatment with DDHAM. No serious or unexpected adverse events were considered related to DDHAM application. The results from this real-world population are compelling and, in many instances, may be considered more useful than “controlled” studies.

### 4.2. Safety

Several of the recently published RCTs evaluated the safety profile of placental-derived biomaterials and concluded that placental-derived biomaterials are safe in the management of hard-to-heal ulcers [132,137,175,180,181,182,184,185,187,190]. Although most investigators did not attribute any adverse events (AEs) to the application of placental-derived biomaterials [132,137,181,184,185,186,187,190], a few were unable to rule out the possibility that an AE was product related [135,189]. In a 2019 study by Tettelbach and colleagues [135], there were 230 AEs reported during the study period. Of these 230, 3 were deemed possibly product related. There was one case of wound maceration and two positive wound cultures. Similarly, in a 2014 study by Serena and colleagues [189], there were 14 AEs reported. Nine AEs occurred in patients treated with dHACM. Five of the nine AEs were unrelated to treatment, but the remaining four were considered potentially product related. There were two cases of cellulitis on the affected extremity: one wound infection and one wound with increased drainage and abscess. In addition, in 2014, Lavery and colleagues conducted a multicenter study comparing the efficacy of a human viable wound matrix (HVWM, Grafix^®^, Osiris Therapeutics, Inc., Columbia, MD) with standard wound care in the treatment of DFUs. Outcome variables included the proportion of AEs and wound related infections. The study found that patients treated with the HVWM had significantly fewer AEs (44% versus 66%) and wound-related infections (18% versus 36%), allowing the authors to conclude that HVWM is a safe treatment for DFUs. The results from RCTs in the past 10 years provide reliable evidence that the placental membrane biomaterials are safe for the management of DFUs and VLUs.

In summary, the available evidence demonstrates that placental-derived biomaterials are a safe and effective means to increase the rate of wound closure compared with conventional wound care alone [192,193]. An increased frequency of application appears to accelerate wound closure [190]; however, additional work is needed to determine the optimal application frequency. Additionally, it is important to note that these study results are generalized. Although the products are correctly classified as placental-derived biomaterials, there are many notable differences, namely, in source, form, preservation, decellularization status, and design. These specific differences have the potential to influence the morphological, physical–chemical, and biological properties of the ECM [6,194] and, perhaps, the clinical effectiveness of the product. Additional RCTs are needed to directly compare the efficacy of different commercially available products in the treatment of complex, hard-to-heal ulcers, considering differences in patient (e.g., age, comorbidities, activity level, and ability to comply with protocol) and wound characteristics (e.g., wound etiology, duration, depth, surface area, exudate, bacterial burden, and location) [116].

## 5. Discussion

Placental-derived biomaterials represent a promising new class of materials for wound healing applications. Placental derivatives have the unique capability of modulating and suppressing innate and adaptive immunities [87,89]. Moreover, placental tissues are a rich source of ECM components, which can be used to create a variety of scaffolds for wound healing.

This review article focused on the use of placental-derived biomaterials as scaffolds in the management of complex, hard-to-heal wounds, such as DFUs and VLUs. Within the last 10 years, reliable evidence has been published demonstrating the safety and effectiveness of placental-derived biomaterials to improve the time to healing. However, there are significant gaps in the literature that require further investigation. Despite the increased market availability of placental-derived scaffolds, it remains unclear how differences in source, preservation techniques, decellularization status, design, and clinical application influence clinical outcomes.

In vitro and clinical evidence supports the application of decellularized placental-derived biomaterials scaffolds in healing [4,5,6,32,148,158]. However, no RCTs have been published comparing decellularized and nondecellularized products in the management of complex wounds. Decellularization of placental-derived biomaterials is performed to limit the immune reaction and inflammatory response induced by the cells, cell debris, and genetic material in the implanted tissue [195]. Decellularized placental tissues have been shown to retain the ECM with a high collagen content (types I, III, and IV) along with key bioactive ECM molecules, such as fibronectin, laminin, glycosaminoglycans, and elastin [99], mimicking the native ECM and creating a reparative environment to promote ECM remodeling, cell migration, proliferation, and differentiation.

Using an in vitro wound healing model, DDHAM (BIOVANCE^®^, Celularity, Florham Park, NJ, USA) was shown to actively direct macrophage polarization into the M2 phenotype [4], mediating a regenerative response and the resolution of wound healing. Biomaterials, capable of directing macrophage polarization, have the potential to restore a timely transition through the phases of wound healing [185,186,187]. Other in vitro models have also demonstrated that decellularized placental-derived biomaterials attenuate the inflammatory response to a greater extent than other nondecellularized placental-derived scaffolds [5,6]. The more pronounced inflammatory response in nondecellularized products may be attributable to the presence of nonviable cells, growth factors, and cytokines [5]. Moreover, the decellularized products appear to promote the activity of various cell types to a greater extent than their nondecellularized counterparts [5,6]. These in vitro reports suggest that decellularized placental-derived biomaterials may more effectively improve healing in clinical applications.

As reviewed Section 4.1, Smiell and colleagues [130] conducted a real-world, multicenter trial to observe the outcomes associated with the use of DDHAM for the treatment of uninfected partial- and full-thickness wounds. Wound management with DDHAM resulted in wound closure after 6.3 weeks, and no DDHAM-related adverse events were reported. DDHAM also achieved wound closure in patients that previously failed one or more advanced biologic therapies. The results from this real-world population demonstrate the effectiveness of DDHAM in treating several wound types.

In 2009, Letendre and colleagues [131] conducted an open-label study to determine the healing rates for partial- and full-thickness DFUs treated with DDHAM. The secondary objective was to determine a safety profile. Of the 14 patients enrolled in the study, 9 patients completed the 12-week study without deviation. All but one wound responded to treatment with DDHAM. A total of 56% of the patients achieved complete wound closure (Group 1), 33% achieved 50–80% wound closure (Group 2), and 11% achieved less than 50% wound closure (Group 3). No adverse events were associated with DDHAM application. These findings demonstrate that DDHAM promotes wound healing and is a safe treatment for partial- and full-thickness DFUs. While the preliminary results evaluating decellularized placental-derived biomaterials show promise, comparative investigations are needed to assess the effect of decellularization status on clinical outcomes.

Moreover, there is a paucity of evidence evaluating placental-derived injectable scaffolds. None of the identified RCTs evaluated placental-derived injectable scaffolds for wound healing. Like sheet scaffolds, placental-derived injectable scaffolds provide structural and biochemical ECM components, but they also have the added benefits of conforming to the contours of irregularly shaped wounds [148,149,153]. This is highly desirable in the treatment of deep tunneling wounds [128]. To date, clinical research is limited. Although case studies have reported the complete epithelialization of complex wounds following treatment with a decellularized flowable placental-derived biomaterial [128,196], RCTs are warranted.

## 6. Next Steps

The use of placental-derived biomaterials in wound healing is a rapidly growing field of research. The existing research establishes placental-derived biomaterials as a safe and effective treatment for the management of complex ulcers. However, additional research is needed to fully understand how the differences in placental-derived biomaterial source, preservation techniques, decellularization methods, design, forms, frequency of application, and methods of administration influence clinical outcomes. Future studies are needed to compare the clinical outcomes associated with the application of decellularized and nondecellularized placental-derived biomaterials in wound management.

## Figures and Tables

**Table 1:** Phases of wound healing. Wound healing is divided into three distinct phases: inflammatory, proliferative, and remodeling.

Phase 1. Inflammatory	Phase 2. Proliferative	Phase 3. Remodeling
Initial Injury–Days 4–6	Day 4–Day 14	Day 8–1 Year
Hemostasis: bleeding stops	Epithelialization	Collagen synthesis
Chemotaxis	Angiogenesis	Collagen turnover
Activation	Granulation tissue formation	Collagen organization

**Table 2:** Factors that impair wound healing.

Local Factors	Systemic Factors
Oxygenation	Age
Infection	Ambulatory status
Venous insufficiency	Comorbidities (e.g., diabetes, obesity, malnutrition, and ischemia)
	Medications (e.g., steroids and nonsteroidal anti-inflammatory drugs)
	Oncology interventions (e.g., radiation and chemotherapy)
	Lifestyle habits (e.g., smoking and alcohol abuse)

**Table 3:** Methods used to decellularize placental tissues.

Method	Agent	Example(s)	Reference(s)
Chemical	Ionic Detergents	SDS SDC Triton-X-200	[2,113,114,115]
	Non-ionic detergents	Triton-X-100	[113,114,115]
	Zwitterionic	CHAPS SB-10 SB-16	[116]
	Acids	Peracetic acid Hydrochloric acid	[117]
	Hypertonic solutions	Sodium chloride	[116,118]
	Hypotonic solutions	Tris-HCl	[116,118]
	Chelating agents	EDTA EGTA	[113,116,119,120]
	Organic solvents	Ethanol Methanol Acetones	[113]
Enzymatic	Proteases	Trypsin	[113]
	Nucleases	DNase RNase	[2,119]
Physical	Pressure	High hydrostatic pressure Supercritical fluids	[5,121,122]
	Temperature	Freeze–thaw	[118,122,123,124]
	Force	Immersion and agitation Shaking Scraping	[2,118,120]

Abbreviations: CHAPS, 3-(3-cholamidopropyl) dimethylammonio-1-proppanesulfonate; DNase, deoxyribonuclease; EDTA, ethylenediaminetetraacetic acid; EGTA, ethylene glycol tetraacetic acid; RNase, ribonuclease; SB-10, sulfobetaine-10; SB-16, sulfobetaine-16; SDC, sodium deoxycholate; SDS, sodium dodecyl sulfate, Tris-HCl, tris-hydrochloride.

**Table 4:** Placental-derived sheet scaffolds for wound healing.

No.	Company	Product	Source	Preservation Method	Decellularization Status	Unique Design Elements	Reference(s)
1	AlloSource	AlloWrap^®^ DS	A	Dry; Proprietary Technology	Nondecellularized	Dual layers, omnidirectional implantation	[155]
2	Amniox Medical, Inc.	Neox^®^ 1K	UC	Cryopreservation; CryoTek^®^	Nondecellularized		
3	Amniox Medical, Inc.	Neox^®^ 100	A	Cryopreservation; CryoTek^®^	Nondecellularized		
4	Amniox Medical, Inc.	Neox^®^ Cord RT	UC & A	Dehydration; SteriTek^®^	Nondecellularized		[140,156,157]
5	Applied Biologics^TM^	Xwrap^®^	A	Dehydration	Nondecellularized	Chorion-free	
6	Celularity Inc.	Biovance^®^	A	Dehydration	Decellularized	Decellularized	[4,32,99,130,131,158]
7	Celularity Inc.	Biovance3L^®^	A	Dehydration	Decellularized	Trilayer	
8	Integra Life Sciences	AmnioExcel^®^	A	Dehydration	Nondecellularized		[7,159,160,161,162,163,164,165,166,167]
9	Integra LifeSciences	BioDFence^®^ G3	A & C	Proprietary method	Nondecellularized	Trilayer (amnion–chorion–amnion)	
10	Integra LifeSciences	BioDDryFlex^®^	A	Dehydration	Nondecellularized		
11	Integra LifeSciences	BioFix^®^	A	Dehydration	Decellularized	Omnidirectional placement	
12	Integra LifeSciences	BioFix^®^ Plus	C	Dehydration; HydraTek^®^	Decellularized	Omnidirectional placement	
13	MiMedx Group, Inc.	EPIFIX^®^	A & C	Dehydration; HydraTek^®^	Nondecellularized	Retains cytokines and growth factors	[129,168]
14	MiMedx Group, Inc.	EPICORD^®^	UC	Dehydration; PURION^®^	Nondecellularized	Expandable	[129,168]
15	MiMedx Group, Inc.	AMNIOCORD^®^	UC	Dehydration; PURION^®^	Nondecellularized	250+ regulatory proteins	[169]
16	MiMedx Group, Inc.	AMNIOEFFECT^®^	A, IL & C	Dehydration; PURION^®^	Nondecellularized	300+ regulatory proteins	
17	MiMedx Group, Inc.	AmnioFix^®^	A & C	Lyophilization; PURION^®^	Nondecellularized	300+ regulatory proteins	[129,168]
18	MTF Biologics	AmnioBand	PT	Dehydration; PURION^®^	Nondecellularized		
19	Organogenesis	Affinity^®^	A	Dehydration; Proprietary aseptic method	Nondecellularized	Fresh allograft derived from amnion tissue	[170,171]
20	Organogenesis	NuShield^®^	A & C	Hypothermically stored	Nondecellularized	LayerLoc™ preserves spongy layer	[134,172,173,174]
21	Skye Biologics, Inc.	WoundEx^®^45	A	Dehydration; LayerLoc™	Nondecellularized	Thin	
22	Skye Biologics, Inc.	WoundEx^®^200	C	Dehydration	Nondecellularized	Thick	
23	Skye Biologics, Inc.	WoundFix™	A	Dehydration	Nondecellularized		
24	Smith & Nephew	GRAFIX^®^ PL	A & C	Dehydration	Nondecellularized	Retains native cells and growth factors	
25	Smith & Nephew	GRAFIX^®^	A & C	Lyopreservation	Nondecellularized	Retains native cells and growth factors	[175,176,177]
26	Smith & Nephew	STRAVIX^®^ PL	UC	Cryopreservation	Nondecellularized		
27	Smith & Nephew	STRAVIX^®^	UC	Lyopreservation	Nondecellularized		[178]
28	StimLabs, LLC	Revita^®^	A, IL & C	Cryopreservation	Nondecellularized		
29	StimLabs, LLC	Cogenex^®^	A, IL & C	Dehydration; Clearify™	Nondecellularized	Fenestrated	
30	StimLabs, LLC	Enverse^®^	A, IL & C	Dehydration	Nondecellularized	Translucent	
31	StimLabs, LLC	Vialize^®^	A, IL & C	Clearify™	Nondecellularized	Lyophilized	
32	Tides Medical	Artacent^®^ Wound	A	Dehydration; Clearify™	Nondecellularized	Dual layer	
33	Ventris Medical	CellestaTM	A	Dehydration; Clearify™	Nondecellularized	Poly mesh backing	
34	Vivex	CYGNUS^®^ Solo	A	Dehydration	Nondecellularized	Single layer	
35	Vivex	CYGNUS^®^ Matrix	A & C	Artacleanse^®^	Nondecellularized		
36	Vivex	CYGNUS^®^ Max	UC	Clearant™	Nondecellularized		
37	Vivex	CYGNUS^®^ Max XL	UC	Dehydrated; INTEGRITY PROCESSING™	Nondecellularized	Fenestrated	

A, amnion; C, Chorion; IL, intermediate layer; PT, placental tissue; UC, umbilical cord.

**Table 5:** Placental-derived injectable scaffolds for wound healing.

#	Company	Product	Source	Preservation Method	Decellularization Status	Unique Design Elements	Reference(s)
1	AediCell	Dermavest^®^/ Plurivest^®^	PD, A, C & UC	Dehydration	Decellularized		
2	Applied Biologics™	FLŌGRAFT^®^	AF	Cryopreservation	Nondecellularized		
3	Celularity Inc.	Interfyl^®^	C	Dehydration	Decellularized	Decellularized	[148]
4	Integra LifeSciences	AmnioMatrix^®^	A & AF	Cryopreservation	Nondecellularized		
5	Integra LifeSciences	BioDFactor^®^	A & AF	Cryopreservation	Nondecellularized		
6	Integra LifeSciences	BioFix^®^ Flo	PT	Dehydration; HydraTek^®^	Decellularized		
7	MiMedx Group, Inc.	AmnioFill^®^	A & C	Dehydration; Purion^®^	Nondecellularized	300+ regulatory proteins	[129]
8	MiMedx Group, Inc.	AxioFillTM	PD	Dehydrated; Purion^®^	Decellularized		
9	Skye Biologics, Inc.	BioRenewTM	PT	Cryopreservation	Nondecellularized	Growth factors	[147]
10	Skye Biologics, Inc.	WoundEx^®^Flow	PT	Dehydration	Nondecellularized		
11	Ventris Medical	CellestaTM Flowable	A	Clearant™	Nondecellularized		

A, amnion; AF, amniotic fluid; C, chorion; IL, PD, placental disc; PT, placental tissue; UC, umbilical cord.

**Table 6:** Randomized controlled trials evaluating placental-derived biomaterials in wound healing.

No.	Study Details	Purpose	Results Summary	Conclusion
1	Study: Mohammadi Tofigh et al., 2022 [180] Study Design: Prospective, Single Center Wound Type: DFU Placental-Derived Biomaterial: dAP (AMOR) Patients: 243 (81 dAP, 81 PDGF Gel, 81 Debridement)	To compare the therapeutic effects of the three methods of diabetic wound care: surgical debridement and dressing, dressing with dAP, and dressing with PDGF gel	Percent area reduction was significantly different among dehydrated amnion, PDGF gel, and debridement at 4 weeks (49.3% vs. 14.8% vs. 7.4%), 6 weeks (79% vs. 35.8% vs. 20.1%), 8 weeks (86.4% vs. 56.8% vs. 43.7%), and weeks 10 and 12 (87.6% vs. 61.7% vs. 50%) Similar safety profiles between groups	Shows improved healing with application of dehydrated amnion powder in DFU patients, compared with platelet-derived growth factor dressing and surgical debridement
2	Study: Serena et al., 2022 [181] Study Design: Prospective, Multicenter Wound Type: VLU Placental-Derived Biomaterial: dHACA (AmnioBand^®^) Patients: 60 (40 dHACA, 20 SOC)	To evaluate the safety and effectiveness of weekly and biweekly applications of dHACA plus SOC compared to SOC alone on chronic VLUs	Significantly higher proportion of healed VLUs in the two dHACA groups than SOC (75% vs. 30%) No significant differences in the proportion of ulcers achieving 40% closure at 4 weeks AE Rate: 63.5%; no graft- or procedure-related AEs	Shows dHACA and SOC, either applied weekly or biweekly, healed significantly more VLUs than SOC alone, suggesting that the use of aseptically processed dHACA is a safe and effective treatment option in the healing of chronic VLUs
3	Study: Game et al., 2021 [182] Study Design: Prospective, Multicenter Wound Type: DFU Placental-Derived Biomaterial: dHAM^®^ (Omnigen) Patients: 31 (15 dHAM, 16 SOC)	To investigate whether 2 weekly additions of the dHAM to standard care versus standard care alone increased the proportion of healed participants’ DFUs within 12 weeks	Similar proportion of healed DFUs for dHAM and SOC (27% vs. 6.3%) Percent wound area reduction was significantly higher in the dHAM group No difference in AEs	Shows dHAM preparation is safe treatment for DFUs
4	Study: Carter, 2020 [183] Study Design: Health Economics Study Wound Type: DFU Placental-Derived Biomaterial: dHACA (AmnioBand^®^) Patients: 80 (40 dHACA, 40 SOC)	To estimate the cost-utility of an aseptically processed dHACA plus SOC versus SOC alone based on a published randomized controlled trial in which patients who had an eligible Wagner 1 DFU wound were randomized to either of these treatments	ICER at 1 year for group 1 versus group 2 was USD-4373 Group 1 had 69.2% lower cost values with increased positive incremental effectiveness for 94.9% of values A willingness to pay curve showed that about 92% of interventions were cost effective for group 1 when USD 50,000 was paid	Demonstrates dHACA added to SOC compared to SOC alone is a cost-effective treatment for DFUs
5	Study: Serena et al., 2020 [171] Study Design: Prospective, Multicenter Wound Type: DFU Placental-Derived Biomaterial: HSAM (Affinity^®^) Patients: 76 (38 HSAM, 38 SOC)	To determine the effectiveness of HSAM versus SOC in DFUs	Proportion of wound closure for HSAM was significantly greater at 12 (55% vs. 29%) and 16 (58% vs. 29%) weeks Incidence of ulcers achieving >60% reductions in area and depth was significantly greater for HSAM (area: 82% vs. 58%; depth: 65% vs. 39%)	Demonstrates an increased frequency and probability of wound closure in DFUs with HSAM versus SOC
6	Study: Tettelbach et al., 2019 [135] Study Design: Prospective, Multicenter Wound Type: DFU Placental-Derived Biomaterial: dHACM (EpiFix^®^) Patients: 110 ITT (54 dHACM, 56 SOC); 98 PP (47 dHACM, 51 SOC)	To confirm the efficacy of dHACM for the treatment of chronic lower extremity ulcers in persons with diabetes	Significantly higher proportion of complete wound closure in 12 weeks for dHACM (ITT: 70% vs. 50%; PP: 81% vs. 55%) A Kaplan–Meier analysis showed a significantly improved time to healing with dHACM Higher proportion of wound remained closed at 16 weeks for dHACM (95% vs. 86%) 230 AEs; 3 possibly product related	Confirms dHACM is an efficacious treatment for lower extremity DFUs
7	Study: Bianchi et al., 2019 [184] Study Design: Prospective, Multicenter Wound Type: VLU Placental-Derived Biomaterial: dHACM (EpiFix^®^) Patients: 128 (64 dHACM, 64 Control)	To report ITT results and assess if both ITT and PP data analyses arrive at the same conclusion of the efficacy of dHACM as a treatment for VLU	Kaplan–Meier plot of time to heal showed a superior wound healing trajectory for dHACM in both ITT and PP Proportion of healed ulcers was significantly greater for dHACM (ITT: 50% vs. 31%; PP: 60% vs. 35%) 65 AEs; none related to dHACM or study procedures	Provides an additional level of assurance regarding the effectiveness of dHACM
8	Study: Tettelbach et al., 2019 [137] Study Design: Prospective, Multicenter Wound Type: DFU Placental-Derived Biomaterial: dHUC (EpiCord^®^) Patients: 155 (101 dHUC, 54 Alginate); 134 PP (86 dHUC; 48 Alginate); 107 AD (67 dHUC; 40 Alginate)	To determine the safety and effectiveness of dHUC allograft compared with alginate wound dressings for the treatment of chronic, nonhealing DFUs	Proportion of patients with complete wound closure at 12 weeks was significantly greater for dHUC (70% vs. 48%) Proportion of AD patients with complete wound closure at 12 weeks was significantly greater for dHUC (96% vs. 65%) Rate of healing at 12 weeks in PP patients was significantly greater for dHUC (81% vs. 54%) 160 AEs; none related to dHUC or alginate dressing	Demonstrates the safety and efficacy of dHUC as a treatment for nonhealing DFUs
9	Study: DiDomenico et al., 2018 [185] Study Design: Prospective, Multicenter Wound Type: DFU Placental-Derived Biomaterial: dHACA (AmnioBand^®^) Patients: 80 (40 dHACA, 40 SOC)	To compare dHACA with SOC in achieving wound closure in nonhealing DFUs	Higher proportion of healed DFUs at 12 weeks for dHACA (85% vs. 33%) Significantly faster mean time to heal for dHACA (37 days vs. 67 days) Mean number of grafts used per healed DFU was 4.0 Mean graft cost per healed DFU was USD 1771 11 AEs; none dHACA related	Shows aseptically processed dHACA heals DFUs significantly faster than SOC at 12 weeks
10	Study: Bianchi et al., 2018 [132] Study Design: Prospective, Multicenter Wound Type: VLU Placental-Derived Biomaterial: dHACM (EpiFix^®^) Patients: 109 (52 dHACM, 57 Control)	To evaluate the efficacy of dHACM as an adjunct to multilayer compression therapy for the treatment of nonhealing full-thickness VLUs	Kaplan–Meier plot of time to heal showed a superior wound healing trajectory for dHACM Proportion of healed ulcers was significantly greater for dHACM at 12 weeks (60% vs. 35%) and 16 weeks (71% vs. 44%) 65 AEs; none related to dHACM or study procedures	Confirms dHACM as an adjunct to multilayer compression therapy for the treatment of nonhealing, full-thickness VLUs
11	Study: Zelen et al., 2016 [186] Study Design: Prospective, Multicenter Wound Type: DFU Placental-Derived Biomaterial: dHACM (EpiFix^®^) Patients: 100 (33 Apligraf, 32 dHACM, 35 SOC)	To compare clinical outcomes at 12 weeks in 100 patients with chronic lower extremity DFUs treated with weekly applications of Apligraf^®^, dHACM, or SOC with collagen–alginate dressing as controls	Significantly higher proportion of healed ulcers for dHACM versus Apligraft versus SOC (97% vs. 73% vs. 51%) Significantly faster time to healing for dHACM versus Apligraft versus SOC (23.6 days vs. 47.9 days vs. 57.4 days) Median number of grafts per healed wound was significantly lower for dHACM (2.5 vs. 6) Median graft cost per healed wound was significantly lower for dHACM (USD 1517 vs. USD 8918) 10 AEs; none dHACM related	Provides further evidence of the clinical and resource utilization superiority of dHACM for the treatment of lower extremity DFUs
12	Study: Zelen et al., 2015 [187] Study Design: Prospective, Multicenter Wound Type: DFU Placental-Derived Biomaterial: dHACM (EpiFix^®^) Patients: 60 (20 Apligraf, 20 dHACM, 20 SOC)	To compare the healing effectiveness of treatment of chronic lower extremity diabetic ulcers with either weekly applications of Apligraf^®^, dHACM, or SOC with collagen–alginate dressing	Significantly higher proportion of complete wound closure for dHACM versus Apligraf and SOC at 4 weeks (85% vs. 35% and 30%) and 6 weeks (95% vs. 45% and 35%) At each week 1–6, mean percent wound size reduction was greatest for dHACM Significantly faster median time to healing for dHACM versus Apligraf and SOC (13 days vs. 49 days and 49 days) Significantly fewer grafts for dHACM (2.15 vs. 6.2) Significantly lower graft cost per patient for dHACM (USD 1669 vs. USD 9216) 5 AEs; none related to treatment	Demonstrates superior clinical and resource utilization for dHACM compared with Apligraf and SOC for the treatment of DFUs
13	Study: Serena et al., 2015 [188] Study Design: Retrospective, Multicenter Wound Type: VLU Placental-Derived Biomaterial: dHACM (EpiFix^®^) Patients: 44 (20 ≥40%, 24 <40%)	To evaluate correct correlation between an intermediate rate of wound reduction (40% wound area reduction after 4 weeks of treatment) and complete healing at 24 weeks in patients with a VLU	Complete healing occurred in 16/20 of the ≥40% group at a mean of 46 days 8/24 of the <40% group at a mean of 103.6 days Correct correlation of status at 4 weeks and ultimate healing status of VLU occurred in 32/44 patients (73%)	Confirms the intermediate outcome is a viable predictor of VLU healing
14	Study: Serena et al., 2014 [189] Study Design: Prospective, Multicenter Wound Type: VLU Placental-derived Biomaterial: dHACM (EpiFix^®^) Patients: 84 (53 dHACM, 31 Control)	To evaluate the safety and efficacy of one or two applications of dHACM and multilayer compression therapy versus multilayer compression therapy alone in the treatment of VLUs	Proportion of patients achieving >40% wound closure at 4 weeks was significantly greater for dHACM (62% vs. 32%). Significant reduction in mean ulcer size for dHACM (48% vs. 19%) 14 AEs (dHACM: 9, Control: 5)	Shows dHACM significantly improved VLU healing at 4 weeks
15	Study: Zelen et al., 2014 [190] Study Design: Prospective, Single Center Wound Type: DFU Placental-Derived Biomaterial: dHACM (EpiFix^®^) Patients: 40 (20 weekly, 20 bi-weekly)	To determine if the weekly application of dHACM allograft reduces time to heal more effectively than biweekly application for treatment of DFUs	Significantly shorter mean time to complete healing in the weekly application group (2.4 ± 1.8 weeks vs. 4.1 ± 2.9 weeks) Proportion of completely healed wounds at 4 weeks was significantly greater in the weekly application group (90% vs. 50%) Similar number of grafts applied to healed wounds (weekly: 2.3 ± 1.8; biweekly: 2.4 ± 1.5) 8 AEs; none attributed to dHACM	Shows dHACM is an effective treatment for DFUs, and DFUs heal more rapidly with weekly application
16	Study: Lavery et al., 2014 [175] Study Design: Prospective, Multicenter Wound Type: DFU Placental-Derived Biomaterial: hVWM (Grafix^®^) Patients: 97 (50 hVWM, 47 Control)	To compare the efficacy of an hVWM with standard wound care to heal DFUs	Proportion of patients with complete wound closure at 12 weeks was significantly greater for hVWM (62% vs. 21%) Significantly shorter median time to healing for hVWM (42 days vs. 69.5 days) Significantly fewer AEs for hVWM (44% vs. 66%) Significantly fewer wound-related infections for hVWM (18% vs. 36%) Similar proportion of wounds remained closed in crossover phase (hVWM: 82%; Control: 70%)	Shows that hVWM is a safe and effective therapy for treating DFUs
17	Study: Zelen et al., 2013 [191] Study Design: Prospective, Single Center Wound Type: DFU Placental-Derived Biomaterial: dHACM (EpiFix^®^) Patients: 25 (13 dHACM, 12 SOC)	To compare healing characteristics of DFUs treated with dHACM versus standard of care	Reductions in wound size were significantly greater for dHACM at 4 weeks (97% vs. 32%) and 6 weeks (98% vs. −2%) Healing rates were significantly higher for dHACM at 4 weeks (77% vs. 0%) and 6 weeks (92% vs. 8%) 5 AEs; none dHACM related	Shows dHACM in addition to the SOC is efficacious for wound healing

Abbreviations: AD, adequate debridement; dAP, dehydrated amnion powder; DFU, diabetic foot ulcer; dHACA, dehydrated human amnion and chorion allograft; dHACM, dehydrated human amnion/chorion membrane; dHAM, dried human amnion membrane; dHUC, dehydrated human umbilical cord; HAA, human amniotic allograft; HSAM, hypothermically stored amniotic membrane; hVWM, human viable wound matrix; ICER, incremental cost-effectiveness ratio; ITT, intent to treat; PDGF, platelet-derived growth factor; PP, per protocol; SOC, standard of care; VLU, venous leg ulcer.

## Data Availability

No new data were created or analyzed in this study. Data sharing is not applicable to this article.
